# The Role of Functional Foods, Nutraceuticals, and Food Supplements in Intestinal Health

**DOI:** 10.3390/nu2060611

**Published:** 2010-06-01

**Authors:** Avrelija Cencic, Walter Chingwaru

**Affiliations:** 1 Department of Microbiology, Biochemistry, Biotechnology and Biotechnology, University of Maribor, Faculty of Agriculture and Life Sciences, Pivola 10, 2311 Hoče, Slovenia; Email: walter.chingwaru@uni-mb.si; 2 University of Maribor, Faculty of Medicine, Slomškov trg 15, 2000 Maribor, Slovenia

**Keywords:** nutraceutical, functional food, food supplement, intestinal health, probiotic, intestinal cell models, gut research

## Abstract

New eating habits, actual trends in production and consumption have a health, environmental and social impact. The European Union is fighting diseases characteristic of a modern age, such as obesity, osteoporosis, cancer, diabetes, allergies and dental problems. Developed countries are also faced with problems relating to aging populations, high energy foods, and unbalanced diets. The potential of nutraceuticals/functional foods/food supplements in mitigating health problems, especially in the gastrointestinal (GI) tract, is discussed. Certain members of gut microflora (e.g., probiotic/protective strains) play a role in the host health due to its involvement in nutritional, immunologic and physiological functions. The potential mechanisms by which nutraceuticals/functional foods/food supplements may alter a host’s health are also highlighted in this paper. The establishment of novel functional cell models of the GI and analytical tools that allow tests in controlled experiments are highly desired for gut research.

## 1. Introduction

Eating habits and trends in food production and consumption have health, environmental and social impacts. Diet has implications on gut health. Gut complications, such as ulcerative colitis, Crohn's disease, irritable bowel syndrome, and gluten therapy resistant celiac, result from overgrowth and imbalance of intestinal microbial flora, and are related to one’s diet. Notably, the gastrointestinal tract is sterile at birth; intestinal microflora develops after birth, with the colonization rate varying according to factors such as the mode of birth, infant nutrition, antibiotic use, diet and age. Gut health determines an individual’s overall health. The human gut has the following functions: (a) it breaks food down to nutrients, (b) it facilitates absorption of nutrients into the blood through intestinal walls, and (c) it prevents foreign and toxic molecules from entering the bloodstream. Gut malfunction, therefore, has a direct negative impact on human health. This review focuses on the role of functional foods, nutraceuticals, and food supplements in intestinal health. 

Consumption patterns vary significantly across Europe, from more southern healthy diets (rich in fruits and vegetables), to the northern ones (rich in animal fat and animal food products). Another striking difference is the fact that in northern European countries, eating is an individual affair, whereas in continental and southern countries consumers attach importance to the social dimension of food and sharing a meal [1]. These nutritional/consumption differences across Europe contribute greatly to the apparent differences in the health of populations on the continent. In spite of the fact that today’s consumers are increasingly attentive to food safety, quality and health-related issues, the population in the European Union (EU) is still fighting with the diseases of a modern age such as obesity, osteoporosis, cancer, diabetes, allergies, stress and dental problems. 

Furthermore, food additives, toxins and emerging food pathogens potentially present in fresh products and processed food present another threat, with their real effects being confounded by their adverse and synergistic effects. On the other hand, the increasingly aging populations in Europe require different foods for healthy aging [2]. Increased energy uptake, unbalanced diets, and highly processed foods used in fast food products are huge problems, which the EU [3] and other developed countries such as the United States of America [4,5,6] have to overcome. Nevertheless, economic crises and high food prices tend to favor unbalanced “cheap” food intake. Attributes such as educational inequalities also contribute to obesity in Europe [7]. However, the USA diet, notably, has the highest amount of calories for the lowest cost worldwide [8]. The USA also has the highest availability of large-scale agricultural commodities, and refined and processed food products. 

Based on the European Food Safety Authority (EFSA) Food-Based Dietary Guidelines, it is evident that in most EU member countries, the most frequent country-specific diet-related health problems are still cardiovascular diseases, overweight/obesity, dyslipidemia, hypertension, type 2 diabetes, osteoporosis and dental caries [9]. In 93% of EU countries, the EFSA reported that the average intake of total fat is above 30% of the energy intake, with the highest intake in Latvia, Lithuania and Slovenia [9]. Only in Italy the intake of saturated fat acids is below 10% [9]. However, overall, females tend to have lower dietary fat and higher carbohydrate intakes than males. Also notable is the fact that more females tend to consume fruits and vegetables within the recommended levels than males in Europe, based on data obtained in Central and Eastern Europe [10]. Moreover, it is a common observation in the EU that among the food groups (bread/cereals, rice/pasta/potatoes, vegetables, fruit, milk/dairy products, meat, fish, oils/fats, legumes and eggs), fruits and vegetables are not prioritized among consumers [11,12]. While consumers are aware of the link between eating and health, they expect visible implications of consumption of a particular product on health [13] and tend to acquire food based on convenience [14]. Recently, food manufacturers have embarked on a health criterion in the development of “functional foods”, the latter being defined as food products that have an added positive health benefit [15]. While some functional ingredient benefits may be perceived to enhance short-term well-being or performance ability, many such benefits concern the long-term mitigation of certain diseases [15]. Long term health benefits are generally invisible to the average consumer. Traditional foods are increasingly considered healthy and wholesome, and as a result, public interest in their nutritional and health impact has increased, as has their demand [15]. 

## 2. Food and Health

Food habits develop in early infancy. In fact, it is known that initial phases of life (intra-uterine period and the first year of life) are sensitive to nutritional factors [16]. Exclusive breastfeeding up to six months, with breastfeeding lasting up to two years or longer in combination with the introduction of balanced complementary feeding (CF), are emphasized by the World Health Organization (WHO) as important measures of public health, with effective impact on the reduction in the risk of developing chronic diseases. It is now known that children after the age of two years should be fed a nutritionally balanced diet similar to that of adults, particularly a diet low in sugar, salt and fat, and rich in complex carbohydrates, fruits and vegetables. Development of food habits is a complex process that may be influenced by such factors as region, religion, family structure and habits, income, prices, stress levels and technological advancements. Several epidemiologic studies over the last 50 years have clearly shown that diets dominated by fruits, vegetables and dietary fibers (plant based foods) prevent and reduce the risk of chronic diseases (e.g. cardiovascular diseases, obesity, diabetes) and promote sound human health. The generation of scientific research linking foods of plant origin and health worldwide has resulted in acknowledgement that plant bioactive compounds have anti-oxidant and other healthy properties [17,18]. 

High dietary intake of fruits, vegetables and whole grains is strongly associated with reduced risk of developing chronic diseases, such as cancer and cardiovascular diseases (CVD), which are the highest causes of death in Europe, United States and in most industrialized countries [9,19]. It is estimated that one-third of all cancer deaths in industrialised countries could be avoided through appropriate dietary formulations. This suggests that dietary behavioral changes, such as increasing consumption of fruits, vegetables, and whole grains, and related changes in lifestyle, are practical strategies for significant reduction of the incidence of cancer [20]. There is a huge amount of scientific literature consistently, but not universally, linking consumption of diets rich in vegetables and fruits with reduced risk of cancer, particularly epithelial cancers of the alimentary and respiratory tracts [21]. Based on results from 206 human epidemiologic studies and 22 animal studies, similar effects of plant based diets are consistent for cancers of the stomach, esophagus, lung, oral cavity and pharynx, endometrium, pancreas, and colon [21]. The types of vegetables or fruit that most often appear to be protective against cancer are raw vegetables, followed by allium vegetables, carrots, green vegetables, cruciferous vegetables, and tomatoes [21].

In 1996 the American Dietetics Association produced a paper demonstrating protective effects of eating fruit and vegetables against several cancers. 

**Cardiovascular diseases** (CVD, including heart disease and stroke), represent the primary cause of death, with high negative impact on both human health and community social costs in Western countries. Cardiovascular diseases and tumors, together, contribute to more than 60 % of deaths in economically-developed countries [22]. In economically-developed countries, CVDs acquire a character of epidemic proportions, and surpass infectious diseases in mortality. Recent studies implicate reactive oxygen species (ROS) in the pathogenesis of both acute and chronic heart diseases as a result of cumulative oxidative stress [23]. In particular, oxidation of low density lipoproteins (LDL), the latter emanating from saturated, trans fats and meat products, has a key role in the pathogenesis of atherosclerosis and cardiovascular heart diseases through the initiation of the plaque formation process [23].

Important risk factors for CVD include obesity, high blood cholesterol level, high blood pressure and type 2 diabetes. The risk of CVD is increased not only by the consumption of poor diets, but also by lifestyle habits such as smoking and alcohol intake [23]. It has been shown that people consuming healthy diets, living active lifestyles, not smoking and not indulging in excessive alcohol consumption tend to have a reduced risk of CVD [24]. It is also known that diets leading to elevated serum total cholesterol, LDL-cholesterol, and triacylglycerol concentrations, while leading to reduced HDL-cholesterol concentrations, lead to reduced risk of coronary artery disease. As a result, treatment of hypercholesterolemia has focused on increasing fecal excretion of cholesterol and bile acids, and reducing hepatic cholesterol synthesis through diet modification among the few optional strategies available [25,26].

Blood pressure control is important in the prevention of heart disease, kidney disease and stroke. Blood pressure is influenced by numerous factors including atherosclerosis, imbalances in the renin-angiotensin system, and hyperinsulinemia, the latter increasing sodium retention in the body and speeding up atherosclerosis [27]. Consequently, a general nutritional plan to minimize hypertensionrisk includes attaining and maintaining a healthy body weight; consuming a diet rich in calcium, phosphorus, and magnesium; and consuming alcoholic beverages and sodium in moderation [28].

**Obesity** is a medical condition characterized by accumulation of excess body fat. As a condition, obesity is associated with reduced life expectancy and/or increased health problems [29]. Obesity is therefore not just a cosmetic problem [29]. Numerous studies indicate that higher levels of body fat are associated with an increased risk of many adverse health conditions. Weight loss is increasingly recognized as bringing major health benefits to overweight people and is linked with increases in life expectancy of people having obesity-related complications [30]. Overweight and obesity have increased over the past 20 years in many regions of the world, particularly the prevalence of obesity in childhood. Obesity is not only restricted to the developed world; it is also becoming a growing burden for the developing countries [31]. Data from the International Obesity Task Force (IOTF) [32] indicate that, worldwide, over 20 million children under the age of six are obese or overweight. Obesity is a multi factorial problem and its development is due to multiple interactions between genes and environment. There is a need to identify aspects of behavior that curtail excessive energy intake while enhancing energy expenditure. These include making appropriate dietary choices, embarking on good eating behavior, and having an active lifestyle [32].

**Cancer** development, a dynamic and long-term process, involves many complex factors with stepwise progression, ultimately leading to an uncontrolled spreading and growth of cancerous cells throughout the body, called metastasis. Epidemiological studies have provided convincing evidence that dietary factors can modify carcinogenesis. Laboratory research has further demonstrated that a number of bioactive dietary components or natural products have the ability to prevent cancer [17,18]. In addition, many food constituents with yet undefined nutritional benefits have been found to possess anti-mutagenic and anti-carcinogenic properties. Such promising research provides a strong support for the acceptance in the future of bioactive components of food as chemopreventative agents [33].

## 3. Functional Foods

Functional foods are similar in appearance to conventional foods; the former being consumed as part of the normal diet. In contrast to conventional foods, functional foods, however, have demonstrated physiological benefits and can reduce the risk of chronic disease beyond basic nutritional functions, including maintenance of gut health [34]. When food is being cooked or prepared using "scientific intelligence" with or without knowledge of how or why it is being used, the food is called "functional food". Thus, functional food provides the body with the required amount of vitamins, fats, proteins, carbohydrates, *etc*., needed for its healthy survival [34].

## 4. Probiotics, Prebiotics and Synbiotics

The human gut is populated by a wide array of bacterial species, the latter bearing important metabolic and immune functions, all leading to marked effects on the nutritional and health status of the host [35]. Probiotics, according to a consensus definition, are ‘living micro-organisms, which upon ingestion in certain numbers, exert health benefits beyond inherent basic nutrition’ [36,37], alternatively probiotics are loosely known as live microorganisms belonging to natural biota with low or no pathogenicity, but with functions of importance to the health and well being of the host [38,39]. Numerous probiotic microorganisms (e.g., *Lactobacillus rhamnosus* GG, *Lactobacillus reuteri*, bifidobacteria and certain strains of *Lactobacillus casei*, the *Lactobacillus acidophilus*-group, *Escherichia coli* strain Nissle 1917, certain enterococci, especially *Enterococcus faecium* SF68, and the probiotic yeast *Saccharomyces boulardii*) are used in probiotic food, particularly fermented milk products, or have been investigated with regard to their medicinal use. New genera and strains of probiotics are emerging; some with high health benefits as in *Lactobacillus plantarum* isolates (PCS20, PCS22, PCS25 and PCS26) from Slovenian cheese with high antimicrobial [40] and immunomodulatory [38] capabilities. Probiotics were originally used to influence human health through intestinal microbiota alterations. At present, probiotics and their effects on human health have been demonstrated both within different food matrices and as single or mixed microbial culture preparations. Furthermore, the health-promoting properties of probiotics are now known to be strain-dependent [37,38,39]. An international expert group of the International Life Sciences Institute (ILSI) has evaluated the categorized and published evidence of functionality of different probiotics in four areas of (human) application, namely, (i) metabolism, (ii) chronic intestinal inflammatory and functionaldisorders, (iii) infections, and (iv) allergy [41]. The ILSI report gives concrete examples demonstrating benefits and gaps, and guidelines and recommendations on the design of next generation of probiotic studies, with the aim to substantiate the current body of information on probiotic benefits. The effects of probiotics on human health are positive and well defined in diarrhoea treatment, however there are no clinical results regarding the dose of probiotics or duration of such treatments [42]. The results from clinical studies have not been conclusive in that the effects of probiotics on the host are dependent on probiotic strains, type of infection (acute or chronic gastrointestinal infections, immunological or inflammatory disease), different doses used and duration of treatment [42]. Probiotics have been shown to have applications in alleviating symptoms of allergies [43,44], cancer [45], AIDS [46], respiratory and urinary tract infections [47]. Furthermore, various findings suggest that probiotics have beneficial effects in alleviating symptoms associated with aging, fatigue, autism, and in reducing the risks of osteoporosis, obesity and possibly type 2 diabetes [48]. Advantages of probiotics for health can only be realized if proper probiotic strain or product selection, and dose guidelines of commercial production, are followed in human food or medicine [44,48].

The concentration of probiotics needed to obtain a clinical effect is often quoted as ≥10^6^ colony forming units (cfu) per milliliter in the small bowel and ≥10^8^ cfu/g in the colon. The dose for treatment of an acute illness by a particular probiotic agent may be lower or higher, in the order of 10-fold or 100-fold or more in terms of cfu. In acute infectious diarrhoea, it seems that higher doses of probiotics given for short courses are more effective than lower doses. In chronic or immunological diseases (allergic, inflammatory and/or immune diseases), the effects of probiotics depend also on the interactions between the respective microorganisms and gut immune system, and duration of treatment. To evaluate the efficacy of probiotics it may be essential to identify specific target groups of individuals with more specific higher susceptibilities to the potential effects of probiotics.

A **prebiotic** is "a selectively fermented ingredient, or a fiber that allows specific changes, both in the composition and/or activity of the gastrointestinal microflora, resultantly conferring benefits on the well being and health of host " [37,48]. Other, more specific effects of prebiotics on health are indirect, namely prevention of diarrhoea or obstipation, modulation of the metabolism of the intestinal flora, cancer prevention, effects on lipid metabolism, stimulation of mineral adsorption and immunomodulatory properties. Prebiotics form a group of diverse carbohydrate ingredients that is poorly understood in regard to their origin, fermentation profiles, and dosages required for health effects, although providing nutraceutical and nutritional value [48]. Today, only bifidogenic, non-digestible oligosaccharides (particularly inulin, its hydrolysis product oligofructose, and (trans)galactooligosaccharides), fulfill all the criteria for prebiotic classification [37]. In the last few years, successful attempts have been reported to make infant formula more breast-milk-like by the addition of fructo- and (primarily) galactooligosaccharides. 

Probiotics and prebiotics share unique roles in human nutrition, largely centered on manipulation of populations or activities of the microbiota that colonize the human GI tract [48]. Regular consumption of probiotics or prebiotics has health implications that include enhanced immune function, improved colonic integrity, decreased incidence and duration of intestinal infections, down-regulated allergic response, and improved digestion and elimination [48]. 

It is noteworthy that human subjects and their enteric microbiota have evolved together to reach a state of mutual tolerance. There is mounting evidence from both animal models and human studies to suggest that inflammatory bowel disease (IBD) is a result of a malfunction of this mutual relationship [49]. Probiotics and prebiotics, however, have been investigated in clinical trials as treatments for IBD, with conflicting results [49]. While there is general consensus about the value of probiotics and prebiotics, their influence on bowl health in terms of Crohn's disease is less convincing [49]. This may be attributed to variations in methodologies, differences in the range of probiotic, prebiotic and combination (synbiotic) treatments tested, and variability in test subjects used, such as different patient groups [49].

**Synbiotics** are synergistic combinations of pro- and prebiotics [37].

### Mechanisms of probiotic actions

While probiotics are gaining significant interest as alternatives for antibiotics or anti-inflammatory drugs, their mode of action is poorly understood [50]. Probiotics may act by modulating the host's immune system, affecting other microorganisms directly or acting on microbial products, host products or food components [50]. The effectiveness of a probiotic depends on its metabolic properties, the set of molecules presented at its surface, the components it secretes, and the integral parts of the microorganism such as its DNA or peptidoglycan [50]. 

### Imbalance of Intestinal Microbial Flora

Modern lifestyles tend to impose stress on systems genetically adapted over millions of years. The consumption of food containing microorganisms has dramatically reduced, and as a consequence, the developing mucosal immune systems are faced with different microflora, particularly fewer pathogens than paleolithic man. Increases in observed incidence and severity of allergies and conditions such as IBD in the Western world have been linked with increases in standards of hygiene and sanitation, which have occurred concomitantly with decreases in the number and range of infectious challenges encountered by the growing and developing host. This lack of immune education impairs the development of the immune system and allows the host to over-react to non-pathogenic antigen-containing commensal flora, resulting in inflammatory damage, allergy and/or autoimmunity [51]. To combat these trends directly, the World Health Organisation currently advocates the implementation of alternative disease control strategies, such as exploiting the prophylactic and therapeutic potential of probiotic bacteria [52]. Most of these probiotic microorganisms, isolated from such sources as faeces of healthy individuals, are safe for human consumption and are available over the counter. Because of continued scepticism of such products, European Union funded research groups including medical, scientific and industrial interests, have agreed on criteria for selection and assessment of probiotics.

## 5. Nutraceuticals

The term "nutraceutical" was coined from "nutrition" and "pharmaceutical" in 1989 by Stephen DeFelice, MD, founder and chairman of the Foundation for Innovation in Medicine (FIM), Cranford, NJ [53]. DeFelice proceeded to define nutraceutical as, "a food (or part of a food) that provides medical or health benefits, including the prevention and/or treatment of a disease" [53]. When functional food aids in the prevention and/or treatment of disease(s) and/or disorder(s) other than anaemia, it is called a nutraceutical [54]. It should be noted that the term nutraceutical, as commonly used in marketing, has no regulatory definition [55]. Thus, nutraceuticals differ from dietary supplements in the following aspects: (1) nutraceuticals must not only supplement the diet but should also aid in the prevention and/or treatment of disease and/or disorder; and (2) nutraceuticals are used as conventional foods or as sole items of a meal or diet [56]. Dietary components play beneficial roles beyond basic nutrition, leading to the development of the functional food concept and nutraceuticals [35]. A functional food for one consumer can act as a nutraceutical for another consumer. Examples of nutraceuticals include fortified dairy products (e.g., milk) and citrus fruits (e.g., orange juice) [56].

Several naturally derived food substances have been studied in cancer therapies. Vitamin E, selenium, vitamin D, green tea, soy, and lycopene are examples of nutraceuticals widely studied in human health [53]. While many of these 'natural' compounds have been found to have high therapeutic potential; future studies should include well-designed clinical trials assessing combinations of these compounds to realize possible synergies they bring into human health. 

Polyunsaturated fatty acids (PUFAs) (which include the omega-3 and omega-6 fatty acids) and phytochemicals also play an important role as healthy dietary bioactive compounds [35]. A balanced PUFA composition of food influences diverse aspects of immunity and metabolism [35]. Moreover, interactions between PUFAs and components of the gut microbiota may also influence their biological roles. Phytochemicals (bioactive non-nutrient plant compounds), have raised interest in human nutrition because of their potential effects as antioxidants, antiestrogenics, anti-inflammatory, immunomodulatory, and anticarcinogenics [17,35]. Gut microbiota can, for example, transform and influence the bioavailability and effects of polyphenols [35]. Phytochemicals and their metabolic products may also inhibit pathogenic bacteria [17,35] while stimulate the growth of beneficial bacteria, exerting prebiotic-like effects [34]. Interactions between functional food components, such as prebiotics, probiotics, phytochemicals, and intestinal microbiota, have consequences on human health [35].

## 6. Food Supplements

According to the US Dietary Supplement Health and Education Act (DSHEA) of 1994, the term "dietary supplement" can be defined using several criteria; namely (a) a product (other than tobacco) that is intended to supplement the diet that bears or contains one or more of the following dietary ingredients: a vitamin, a mineral, an herb or other botanical, an amino acid, a dietary substance for use by man to supplement the diet by increasing the total daily intake, or a concentrate, metabolite, constituent, extract, or combinations of these ingredients [55], (b) a product intended for ingestion in pill, capsule, tablet, or liquid form, (c) a product not represented for use as a conventional food or as the sole item of a meal or diet, (d) anything labeled as a "dietary supplement", and (e) products such as a newly approved drug, certified antibiotic, or licensed biologic that was marketed as a dietary supplement or food before approval, certification, or license (unless if this provision is waived by an authority such as Secretary of Health and Human Services, as in USA) [56].

Under the DSHEA (1994), the manufacturer of a dietary supplement is responsible for ensuring that the dietary supplement is safe before it is marketed [57]. It is also binding to the manufacturers that they make sure that product label information is truthful and not misleading [57].

Many European countries have adopted the highly restrictive CODEX standards for dietary supplements, which eliminate the consumer’s ability to purchase dietary supplements in therapeutic or meaningful preventive dosages. Codex Alimentarius (Latin for "Food Code") is the United Nation’s proposed set of international guidelines for nutritional supplements, food handling, production, and trade which is gradually being ratified in countries around the world, starting in the EU [58]. In comparison to most other industrialized countries, there is virtually no restriction on the type or strength of nutritional supplements that can be purchased in the United States [58]. The only significant limitation on the sale of supplements is that disease and cure related claims cannot be made by supplement manufacturers without USA Food and Drug Agency (FDA) approval. And the FDA has approved only a few disease related claims for supplements [58].

Questions can arise whether a nutrient used as part of a treatment for a defined disease can be considered a drug, whereas the same nutrient is used to enhance health (reduce the risk of disease), can be considered a functional food or dietary supplement? This overlap shows interlinks that exist between functional foods and medicines.

Mounting evidence supports the observation that functional foods containing physiologically active components, either from plant or animal sources, may enhance health [59]. Clearly, all foods are functional, as they provide taste, aroma, or nutritive value [59]. There has been an explosion of consumer interest in the health enhancing role of specific foods or physiologically-active food components, so-called functional foods [59]. It should be stressed, however, that functional foods are not a magic bullet or universal panacea for poor health habits [59]. There are no "good" or "bad" foods, but there are good or bad diets [59]. 

## 7. The Intestinal Health

Proper functioning of the digestive tract, with both its chemical and muscular activity, is essential to health. Digestive disorders are on the increase. About 38 million Americans suffer from a variety of digestive problems such as gastroesophageal reflux disease (GERD), irritable bowel syndrome, celiac disease, food allergies, diverticulitis, ulcerative colitis, and Crohn’s disease [60]. Approximately 25 million Americans have daily heartburn, and it is estimated that 20% of the adult population have irritable bowel syndrome. Celiac disease, once considered rare, is now thought to affect 1 in 133 people, and food allergies have increased alarmingly [60].

## 8. The Gastrointestinal (GI) Tract as an Ecosystem

Co-evolution led to a symbiotic relationship between eukaryotes and prokaryotes with the development of sophisticated by-directional signaling systems of mucosal epithelia and lymphocytes in the intestinal tract [51]. It is estimated that over 400 species of bacteria, separated into two broad categories, namely beneficial (e.g., *Bifidobacterium* and *Lactobacillus*) and those considered detrimental (e.g., Enterobacteriaceae and *Clostridium* spp.) inhabit the human gastrointestinal tract. Bacterial end products of fermentation are essential mucosal nutrients including amino acids (arginine, cysteine and glutamine) and short chain fatty acids (SCFA: acetate, propionate and butyrate) [51]. These SCFAs serve as an energy source for the host, providing 10–30% of basal metabolic requirements including energy for liver cells, colonocytes and peripheral tissues with only about 5% excreted in the feces [51]. Besides fermentation, the metabolic products of the microflora includes vitamins K and B complex, secondary bile acid production, neutralization of dietary carcinogens such as nitrosamines, and conversion to active metabolites of some prodrugs. The indigenous intestinal (autochthonous) microbiota act as a further barrier against any transient (allochthonous) potential pathogens by competing for nutrients and mucosal adherence and by production of antigens (bacteriocins), which are active against pathogens. Furthermore, it has been clearly established that the gastrointestinal flora are essential for mucosal protection and immune education as it has been described as the most adaptable and renewable metabolic organ of the body. The composition and activities of gastrointestinal flora affect both intestinal and systemic physiology. The complex gastrointestinal microbial load is required for normal development and homeostasis of the humoral and cellular immune system. It is the interaction between the mucosal immune system and the enteric microflora which maintains the physiologically normal state or activation of immune organ, the latter resulting in secretion of antibodies against harmful antigens (pathogenic microorganisms) [51]. 

## 9. Application of Intestinal Cell Models

Functional cell/co-culture models involve growth of cell lines in co-culture, either in direct contact with each other on the membrane or indirectly where one cell line is cultured on the apical side of a membrane and the other cell line on either the underside of the membrane (basolateral) or on the surface of the plastic feeder or receiver trays in multi-well cell plates [61,62,63,64]. Use of cells in co-culture systems mimics the natural existence of such cells in an organism, so that direct effects on cells, tissues or organs of chemicals, microorganisms and their products and so forth, can be tested *in situ*. The direct mixing of different cell types in culture is otherwise normally avoided in co-culture systems, allowing scientists to model such interactions *in vivo*. Functional/co-culture cell models are increasingly gaining recognition in science, as they mimic normal organ, tissue and physiological organization in human organism. 

In a co-culture system, two or more cell types brought together in the same culture environment very likely interact and communicate [65]. Co-culture has proved to be a powerful *in vitro* tool in unravelling the importance of cellular interactions during normal physiology, homeostasis, repair and regeneration [65]. 

**Figure 1:**
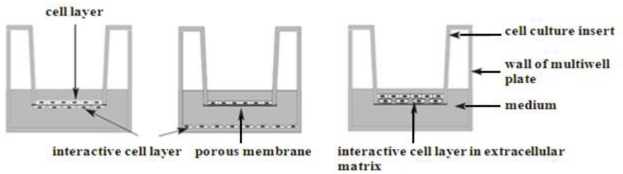
Co-culture set up system.

Co-culture cell systems have been used in a variety of applications such studies on disease pathogenesis, for example Crohn’s disease [63] drug testing [64], study of xenobiotic kinetics [66], studies on cancer progression [67], to name a few examples. The advantages of studying effects of different substances *in vitro* on cell lines include: (i) the ability to manipulate cells in a defined environment and to retrieve and study specific cell types; (ii) ability to identify novel growth or inhibitory paracrine factors involved in regulation of growth; (iii) cell behavior is easier to reproduce in these *in situ* models than *in vivo*; (iv) they provide a physiological basis for future studies of molecular and genetic mechanisms of disease; and (v) they allow dissection of mechanisms of cell-cell and cell-matrix interactions involved in human disease, while avoiding extrapolation of data from studies using other species. Co-culture systems are therefore important developments with novel medical applications and resulting in reduced testing of substances on living animals. 

### Co-culture systems and studies of interactions between probiotic bacteria and intestinal epithelium

Recently Gradisnik *et al*., (2006) [68] reported establishment of a pig functional cell model involving two pig intestinal epithelial cell lines (PSI and CLAB) and macrophages from pig peripheral blood (PoMac), with possible applications in both human and animal studies. This model was developed in sight of the substantial lack of good intestinal cell culture models. It was noted that pig cell lines represented better animal models than rodents in human research. Recently Nissen *et al*., (2009) [39] have reported use of the pig functional cell model described by Gradisnik *et al*. [68] in studies of interactions between intestinal epithelium, macrophages and probiotic bacteria. Such a model has been found to give novel effects in such interactions. Current efforts are centred on development of human co-culture model systems, including use of three-dimensional co-culture systems for use in studying gut health, and development of nutraceuticals or functional ingredients of foods with useful protective effects on the GI tract. 

## 10. Conclusion

Overall, this paper discusses nutraceuticals/functional foods/food supplements (broadly including probotics, prebiotics, synbioitcs, phytochemicals *etc.*), especially the need for consuming appropriate diets, health issues surrounding failure to adhere to the known healthy eating models, development of new nutraceuticals/functional foods/food supplements with novel health benefits, elucidation mechanisms of action of these products, development of study systems such as *in-vitro* co-culture cell models. Appropriate diet culminates in a healthy, properly functioning GI tract, resulting in attainment of proper human physiology, hence healthy living; otherwise the opposite becomes true. Modeling new eating habits using the existing knowledge is needed for the eventual ideal of ‘health for all’ vision. 

## References

[1] Bronzwaer S. (2008). EFSA scientific forum “from safe food to healthy diets”. EU risk assessment -Past, present and Future. Trends Food Sci. Technol..

[2] Roberts S.B., Rosenberg I. (2006). Nutrition and aging: changes in the regulation of energy metabolism with aging. Physiol. Rev..

[3] Perez-Cueto F.J., Verbeke W., de Barcellos M.D., Kehagia O., Chryssochoidis G., Scholderer J., Grunert K.G. (2009). Food-related lifestyles and their association to obesity in five European countries. Appetite.

[4] Gilbert R.L., Mielke J.H. (1985). The Analysis of Prehistoric Diets.

[5] Eaton S.B., Eaton S.B., Konner M.J., Shostak M. (1996). An evolutionary perspective enhances understanding of human nutritional requirements. J. Nutr..

[6] Eaton S.B., Eaton S.B., Konner M.J. (1997). Paleolithic nutrition revisited: a twelve-year retrospective on its nature and implications. Eur. J. Clin. Nutr..

[7] Roskam A.J., Kunst A.E., Van Oyen H., Demarest S., Klumbiene J., Regidor E., Helmert U., Jusot F., Dzurova D., Mackenbach J.P. (2009). Comparative appraisal of educational inequalities in overweight and obesity among adults in 19 European countries. Int. J. Epidemiol..

[8] Crawford M.A., Marsh D. (1995). Nutrition and Evolution.

[9] EFSA (European Food Safety Authority) (2008). Scientific Opinion of the Panel on Dietetic Products, Nutrition and Allergies on a request from the EC on Food-Based Dietary Guidelines. The EFSA J..

[10] Boylan S., Welch A., Pikhart H., Malyutina S., Pajak A., Kubinova R., Bragina O., Simonova G., Stepaniak U., Gilis-Januszewska A., Milla L., Peasey A., Marmot M., Bobak M. (2009). Dietary habits in three Central and Eastern European countries: the HAPIEE study. BMC Public Health..

[11] Acheson D. (2002). Independent Inquiry into Inequalities in Health, Foodaware Proposals for an EU Nutrition Policy, Comments on the Joint WHO/FAO Expert Consultation on Diet, Nutrition and the Prevention of Chronic Diseases. http://www.who.int/dietphysicalactivity/media/en/gsfao_cmo_035.pdf.

[12] NHS (2008). Exploration of Adult Food Portion Size Tools.

[13] Brunso K., Fjord T.A., Grunert K.G. (2002). Consumers' food choice and quality perception.

[14] Grunert K.G., Brunso K., Bredahl L., Bech A.C., Frewer L.J., Risvik E., Schifferstein H.N.J., von Alvensleben R. (2001). Food-Related Lifestyle: A Segmentation Approach to European Food Consumers. Food, People and Society: A European Perspective of Consumers’ Food Choices.

[15] (1998). Public Health Boon or 21^st^ Century Quackery? International, Functional Foods, Center for Science in the Public. CSPI Reports.

[16] Caetano M.C., Ortiz T.T., da Silva S.G., de Souza F.I., Sarni R.O. (2010). Complementary feeding: inappropriate practices in infants. J. Pediatr. (Rio J)..

[17] Cencic A., Chingwaru W. (2010). Antimicrobial Agents Deriving from Indigenous Plants. RPFNA.

[18] Balsano C., Alisi A. (2009). Antioxidant effects of natural bioactive compounds. Curr. Pharm. Des..

[19] Liu R.H. (2004). Potential synergy of phytochemicals in cancer prevention: mechanism of action. J. Nutr..

[20] Terry P., Giovannucci E., Michels K.B., Bergkvist L., Hansen H., Holmberg L., Wolk A. (2001). Fruit, vegetables, dietary fiber, and risk of colorectal cancer. J. Natl. Cancer. Inst..

[21] Denny A., Buttriss J. (2005). Synthesis Report No 4: Plant Foods and Health: Focus on Plant Bioactives. http://www.eurofir.net/temp/PLANTspFOODSspANDspHEALTHspFOCUSspONspPLANTspBIOACTIVEShs1hs.pdf.

[22] Stramba-Badiale M., Fox K.M., Priori S.G., Collins P., Daly C., Graham I., Jonsson B., Schenck-Gustafsson K., Tendera M. (2006). Cardiovascular diseases in women: a statement from the policy conference of the European Society of Cardiology. Eur. Heart. J..

[23] Wang C.Z., Mehendale S.R., Yuan C.S. (2007). Commonly used antioxidant botanicals: active constituents and their potential role in cardiovascular illness. Am. J. Chin. Med..

[24] Riccioni G., Mancini B., Di Ilio E., Bucciarelli T., D'Orazio N. (2008). Protective effect of lycopene in cardiovascular disease. Eur. Rev. Med. Pharmacol. Sci..

[25] The Expert Panel (1993). Summary of the second report of the National Cholesterol Education Program (NCEP) Expert Panel on detection, evaluation, and treatment of high blood cholesterol in adults (Adult Treatment Panel II). JAMA.

[26] Austin M.A. (1991). Plasma triglyceride and coronary heart disease. Arterioscler. Thromb..

[27] Lampe J.W. (1999). Health effects of vegetables and fruit: assessing mechanisms of action in human experimental studies. Am. J. Clin. Nutr..

[28] Dwyer J. (1995). Overview: dietary approaches for reducing cardiovascular disease risks. J. Nutr..

[29] Haslam D.W., James W.P. (2005). Obesity. Lancet.

[30] Rayalam S., Della-Fera M.A., Baile C.A. (2008). Phytochemicals and regulation of the adipocyte life cycle. J. Nutr. Biochem..

[31] Martorell R., Kettel Khan L., Hughes M.L., Grummer-Strawn L.M. (2000). Overweight and obesity in preschool children from developing countries. Int. J. Obes. Relat. Metab. Disord..

[32] International Obesity TaskForce (IOTF) (2005). Obesity in Europe, EU Platform Briefing Paper, prepared by Lobstein, T.; Rigby, N.; in collaboration with European Association for the Study of Obesity. http://www.iaso.org/popout.asp?linkto=http%3A//www.iotf.org/media/euobesity3.pdf.

[33] Pan M.H., Ghai G., Ho C.T. (2008). Food bioactives, apoptosis, and cancer. Mol. Nutr. Food. Res..

[34] Food and Agriculture Organization of the United Nations (FAO) (2007). Report on Functional Foods, Food Quality and Standards Service (AGNS). http://www.fao.org/ag/agn/agns/files/Functional_Foods_Report_Nov2007.pdf.

[35] Laparra J.M., Sanz Y. (2010). Interactions of gut microbiota with functional food components and nutraceuticals. Pharmacol. Res..

[36] Guarner F., Schaafsma G.J. (1998). Probiotics. Int. J. Food Microbiol..

[37] de Vrese M., Schrezenmeir J. (2008). Probiotics, prebiotics, and synbiotics. Adv. Biochem. Eng. Biotechnol..

[38] Bengmark S. (1998). Ecological control of the gastrointestinal tract. The role of probiotic flora. Gut..

[39] Nissen L., Chingwaru W., Sgorbati B., Biavati B., Cencic A.  (2009). Gut health promoting activity of new putative probiotic/protective Lactobacillus spp. strains: a functional study in the small intestinal cell model. Int. J. Food. Microbiol..

[40] Maragkoudakis P.A., Chingwaru W., Gradisnik L., Tsakalidou E., Cencic A. (2010). Lactic acid bacteria efficiently protect human and animal intestinal epithelial and immune cells from enteric virus infection. Int. J. Food. Microbiol..

[41] Rijkers G.T., Bengmark S., Enck P., Haller D., Herz U., Kalliomaki M., Kudo S., Lenoir-Wijnkoop I., Mercenier A., Myllyluoma E., Rabot S., Rafter J., Szajewska H., Watzl B., Wells J., Wolvers D., Antoine J.M. (2010). Guidance for substantiating the evidence for beneficial effects of probiotics: current status and recommendations for future research. J. Nutr..

[42] Minelli E.B., Benini A. (2008). Relationship between number of bacteria and their probiotic effects. Microb. Ecol. Health Dis..

[43] Yao T.C., Chang C.J., Hsu Y.H., Huang J.L. (2009). Probiotics for allergic diseases: Realities and myths. Pediatr. Allergy Immunol..

[44] Kalliomaki M., Antoine J.M., Herz U., Rijkers G.T., Wells J.M., Mercenier A. (2010). Guidance for substantiating the evidence for beneficial effects of probiotics: prevention and management of allergic diseases by probiotics. J. Nutr..

[45] Kumar M., Kumar A., Nagpal R., Mohania D., Behare P., Verma V., Kumar P., Poddar D., Aggarwal P.K., Henry C.J., Jain S., Yadav H.  (2010). Cancer-preventing attributes of probiotics: an update. Int. J. Food. Sci. Nutr..

[46] Trois L., Cardoso E.M., Miura E. (2008). Use of probiotics in HIV-infected children: a randomized double-blind controlled study. J. Trop. Pediatr..

[47] Kaur I.P., Kuhad A., Garg A., Chopra K. (2009). Probiotics: delineation of prophylactic and therapeutic benefits. J. Med. Food..

[48] Douglas L.C., Sanders M.E. (2008). Probiotics and prebiotics in dietetics practice. J. Am. Diet. Assoc..

[49] Hedin C., Whelan K., Lindsay J.O. (2007). Evidence for the use of probiotics and prebiotics in inflammatory bowel disease: a review of clinical trials. Proc. Nutr. Soc..

[50] Oelschlaeger T.A. (2010). Mechanisms of probiotic actions -A review. Int. J. Med. Microbiol..

[51] O'Sullivan G.C., Kelly P., O'Halloran S., Collins C., Collins J.K., Dunne C., Shanahan F. (2005). Probiotics: An Emerging Therapy. Curr. Pharm. Design.

[52] Dunne C., O'Mahony L., Murphy L., Thornton G., Morrissey D., O'Halloran S., Feeney M., Flynn S., Fitzgerald G., Daly C., Kiely B., O'Sullivan G.C., Shanahan F., Collins J.K. (2001). *In vitro* selection criteria for probiotic bacteria of human origin: correlation with *in vivo* findings. Am. J. Clin. Nutr..

[53] Brower V. (1998). Nutraceuticals: poised for a healthy slice of the healthcare market?. Nat. Biotechnol..

[54] Trottier G., Bostrom P.J., Lawrentschuk N., Fleshner N.E. (2010). Nutraceuticals and prostate cancer prevention: a current review. Nat. Rev. Urol..

[55] Zeisel S.H. (1999). Regulation of "Nutraceuticals". Science.

[56] Kalra E.K. (2003). Nutraceutical -Definition and Introduction. AAPS PharmSci..

[57] (2006). The Regulation of Dietary Supplements. United States Pharmacopeia (USP).

[58] Hasler C.M. (1998). Functional Foods: Their role in disease prevention and health promotion, Institute of Food Technologists’ Expert Panel on Food Safety and Nutrition. Foodtechnology..

[59] Gaffe R. (2010). The Current and Future Regulation of Dietary Supplements. http://www.richardjaffeesq.com/jaffe/dietarysupplements.asp.

[60] Biddle J., Dasgupta-O’Brien S., Walch A. Gut Health, Asheville Integrative Medicine (undated). http://www.docbiddle.com/moreinfo/guthealth.pdf.

[61] Mangum J.B., Everitt J.I., Bonner J.C., Moore L.R., Brody A.R. (1990). Co-culture of primary pulmonary cells to model alveolar injury and translocation of proteins. In Vitro Cell. Dev. Biol..

[62] Arnold J.T., Kaufman D.G., Seppala M., Lessey B.A. (2001). Endometrial stromal cells regulate epithelial cell growth *in vitro*: a new co-culture model. Hum. Reprod..

[63] Willemsen L.E., Schreurs C.C., Kroes H., Spillenaar Bilgen E.J., Van Deventer S.J., Van Tol E.A. (2002). A co-culture model mimicking the intestinal mucosa reveals a regulatory role for myofibroblasts in immune-mediated barrier disruption. Dig. Dis. Sci..

[64] Depasquale I., Wheatley D.N. (2006). Action of Lovastatin (Mevinolin) on an *in vitro* model of angiogenesis and its co-culture with malignant melanoma cell lines. Cancer Cell Int..

[65] Hendriks J., Riesle J., van Blitterswijk C.A. (2007). Co-culture in cartilage tissue engineering. J. Tissue Eng. Regen. Med..

[66] Bhatia S.N., Balis U.J., Yarmush M.L., Toner M. (1999). Effect of cell-cell interactions in preservation of cellular phenotype: cocultivation of hepatocytes and nonparenchymal cells. The FASEB J..

[67] Brillet B., Petiot S., Iochmann S., Gaud G., Planque C., Blechet C., Heuze-Vourc’h N., Gruel Y., Courty Y., Reverdiau P. (2008). 025 Tumor-stromal cell interactions modulate metalloproteinase and kalli Krein expression in direct and indirect co-culture cell models. Revue des Maladies Respiratoires.

[68] Gradisnik L., Filipic B., de Vaureix C., Lefevre F., La Bonnardiere C., Cencic A. (2006). Establishment of a functional cell culture model of the pig small intestine, LINZ, ALTEX 23. ALTEX Altern. Tierexp..

